# Successful Reconstruction of Tooth Germ with Cell Lines Requires Coordinated Gene Expressions from the Initiation Stage

**DOI:** 10.3390/cells1040905

**Published:** 2012-10-30

**Authors:** Akihiko Komine, Yasuhiro Tomooka

**Affiliations:** Department of Biological Science & Technology, Science University of Tokyo, 2641 Yamazaki Noda, Chiba 278-8510, Japan

**Keywords:** tooth reconstruction, clonal cell lines, reprograming, dental epithelial cells, initiation stage

## Abstract

Tooth morphogenesis is carried out by a series of reciprocal interactions between the epithelium and mesenchyme in embryonic germs. Previously clonal dental epithelial cell (epithelium of molar tooth germ (emtg)) lines were established from an embryonic germ. They were odontogenic when combined with a dental mesenchymal tissue, although the odontogenesis was quantitatively imperfect. To improve the microenvironment in the germs, freshly isolated dental epithelial cells were mixed with cells of lines, and germs were reconstructed in various combinations. The results demonstrated that successful tooth construction depends on the mixing ratio, the age of dental epithelial cells and the combination with cell lines. Analyses of gene expression in these germs suggest that some signal(s) from dental epithelial cells makes emtg cells competent to communicate with mesenchymal cells and the epithelial and mesenchymal compartments are able to progress odontogenesis from the initiation stage.

## 1. Introduction

The dental morphogenesis is regulated by interactions between the dental epithelium and cranial neural crest-derived ectomesenchyme; the elongated inner enamel epithelium (ameloblast) secretes enamel and the elongated dental mesenchyme (odontoblast) secretes dentin. Various signal factors mediate the interactions at all stages of the dental development [1,2]. The dental epithelium has various types of cells and its population changes as the dental germ develops.

To investigate the dental morphogenesis, cell lines have been established with SV40 transfection or spontaneous immortalization of dental epithelial cells [3,4,5] and of dental mesenchymal cells [6,7,8]. However, no attempts have reported on successful reconstruction of teeth with epithelial cell lines. We have reported that clonal cell lines can be established by a simple dilution culture method from various tissues of *p53*-deficient mice, such as reproductive tissues [9,10,11,12,13] and neural tissues [14,15,16,17]. Based upon these studies, we have recognized two critical points: (1) clonal and immortalized cell lines express the tissue specific markers *in vitro* when they are established from adult tissues, and (2) cell lines preserve developmental or transient features when they are established from fetal tissues. We then established clonal cell lines from a dental epithelium of a molar tooth germ and demonstrated that they can reconstruct well-calcified teeth [18].

Recent studies indicate that the microenvironment in some tissues reprograms the already-made program in foreign cells and allows them to take its native program [19,20,21]. When bone marrow cells were mixed with dental epithelial cells prepared from embryos, and a dental germ was reconstructed with the mixed cells and dental mesenchyme, the germ developed a tooth. The mixed bone marrow cells differentiated into ameloblasts in the tooth [19]. It is unknown, however, how the microenvironment induced the transdifferentiation. The microenvironment in a developmental tissue seems to be plastic. Previous studies demonstrated that reassociation of the epithelium and mesenchyme separated from a germ restarted developmental events from the initiation stage [22,23,24,25,26]. These studies indicate that (1) a developmental program proceeding in a germ is canceled when the epithelial and mesenchymal compartments are artificially separated, (2) when the compartments are recombined, they reorganize a germ in which the program is reinitiated, and (3) if foreign cells are involved in the epithelial compartment at reinitiation, the microenvironment cancels the already-made program in the foreigners and allows them to take its own program. 

In the present study, we adopted the paradigm of reconstruction of dental germs with emtg cell lines established from an embryonic dental germ. Cells of emtg lines and epithelial cells isolated from dental germs were mixed and germs were reconstructed with the mesenchymal tissues. Tooth morphogenesis was observed in the germs cultured *in vivo* and *in vitro*. The results revealed how the microenvironment in the germs had influences on tooth construction and how the microenvironment was created.

## 2. Results and Discussion

### 2.1. Results

#### 2.1.1. Odontogenesis of Emtg-2 Cells Was Improved by Embryonic Dental Epithelial Cells (*in Vivo*)

Clonal epithelial cell lines (emtg-1 to -5) were established from a mouse molar tooth germ. Germs reconstructed with cells of lines and molar mesenchymal tissues developed teeth when transplanted under kidney capsule, although tooth construction rate was below 100% [18]. 

**Figure 1:**
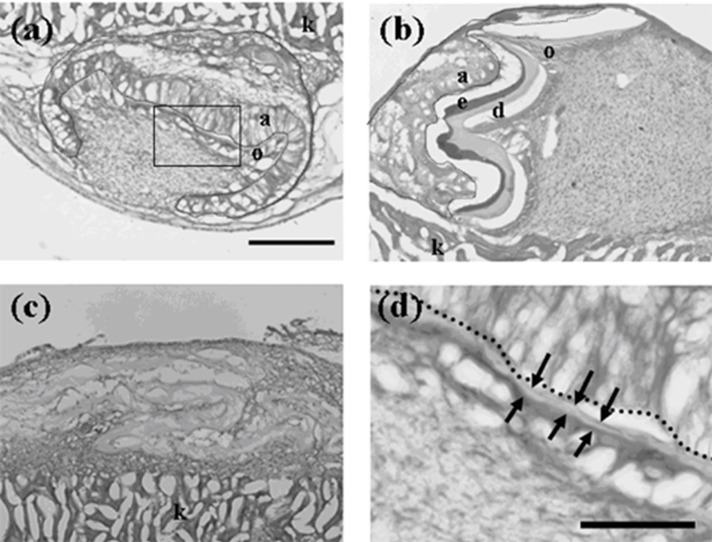
Histology of constructed teeth from germs implanted under kidney capsule. (**a**) A tooth developed from a germ reconstructed with mixed epithelial cells (emtg-2 cells: dental epithelial cells = 1:1) and a dental mesenchymal tissue (MT). (**b**) A constructed tooth with dental epithelial cells (EC) and MT prepared from a first molar (E16.5) (positive control). (**c**) Bone-like tissues were developed from a germ reconstructed with only MT (negative control). (**d**) High magnification of the enclosed area in (**a**). The development of the tooth (**a**) was delayed because it had a thinner calcified layer than that of (**b**). Nuclei were stained with DAPI (blue). a: elongated inner enamel epithelium (ameloblast-lineage), d: dentin, e: enamel, o; odontoblast, dot line; dental epithelium, arrows; thin dentin layer, k; kidney. Scale bar is 200 μm in (a, b and c), 50 μm in (**d**).

The previous study has raised the question of whether or not the microenvironment in the reconstructed germs is poorly created. In a reconstructed tooth germ, a pellet of epithelial cells (emtg cells) is sitting on a mesenchymal tissue (MT) prepared from a lower first molar germ (E16.5). To improve the microenvironment, germs were reconstructed with a mixture of emtg-2 cells and epithelial cells prepared from a lower first molar germ (EC, E16.5) (mixing ratio; emtg-2 cells: EC = 1:1) and with MT, and implanted under kidney capsule for 7 days. The germs developed teeth at 100% (Figure 1a and Table 1), as observed in positive controls (mixing ratio; emtg-2 cells: EC = 0:1, with MT) (Figure 1b, and Table 1). No tooth structures developed from germs reconstructed without epithelial cells (negative control) (Figure 1c, Table 1). Active involvement of emtg-2 cells in the odontogenesis is shown in 2.1.5

**Table 1:** Tissues grown from implanted tooth germs (emtg-2: dental epithelial cells [EC] = 1:1).

	Tooth	Non-Tooth	Total
Positive control	7	−	7
Negative control		9	9
emtg-2:EC = 1:1	6	−	6

#### 2.1.2. Odontogenesis of Emtg-2 Cells on Culture Inserts

**Figure 2:**
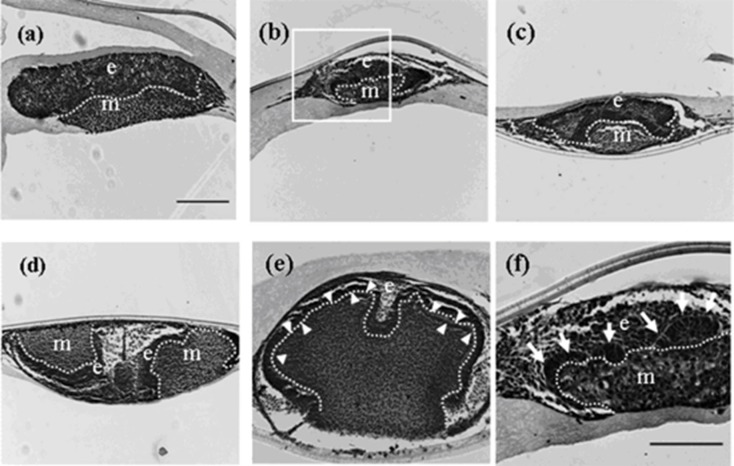
Histology of developing germs reconstructed with mixed epithelial cells (emtg-2 cells: EC = 1:1) and MT on culture inserts. Germs cultured for 1 (**a**), 2 (**b**), 3 (**c**), 5 (**d**) and 7 days (**e**). (**f**) High magnification of the enclosed area in (**b**). Elongated epithelial cells (arrows in **f**) were detected after two days in culture. A developing tooth had a very thin dentin-like structure after seven days in culture (e: arrow heads). E: epithelium, m; mesenchyme, dot line; epithelial-mesenchymal interface. Scale bar in (**a**) is 200 μm and figure b–e are at the same magnification. Scale bar in (**f**) is 100 μm.

**Figure 3:**
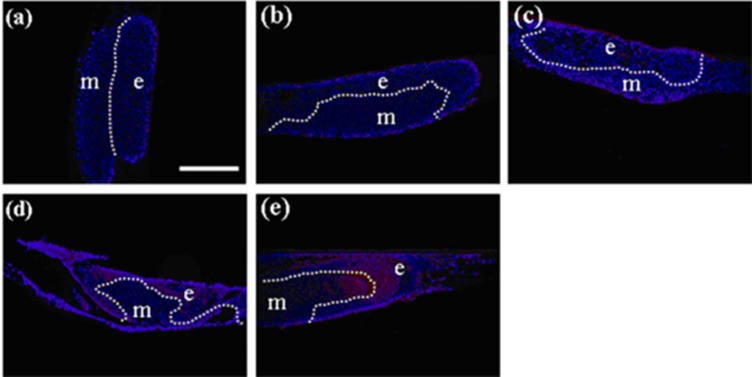
Immunohistochemistry of developing teeth from germs reconstructed with mixed epithelial cells (emtg-2 cells: EC = 1:1) and MT on culture inserts. Germs were cultured for 1 (**a**), 2 (**b**), 3 (**c**), 5 (**d**) and 7 days (**e**). Amelogenin protein (red) became detectable after seven days in culture (**e**). Nuclei were stained with DAPI (blue). E: epithelium, m; mesenchyme, dot line; epithelial-mesenhymal interface. Scale bar is 200 μm in (**a**) and all figures are at the same magnification.

Germs reconstructed with EC and MT developed teeth when cultured on cell culture inserts [22]. Therefore, germs were reconstructed with mixed epithelial cells (emtg-2 cells: EC = 1:1) and with MT, and were cultured on inserts. It was confirmed that all germs developed teeth on inserts (Figure 2 and Table 2) as observed in positive controls (emtg-2 cells: EC = 0:1, data not shown). Elongated epithelial cells like those in the inner enamel epithelium (IEE) were detected after two days in culture (Figure 2b,f) and a very thin dentin-like structure was detected after seven days in culture (Figure 2e). Immunohistochemistry confirmed amelogenin expression in the elongated epithelial cells adjacent to the mesenchyme in germs after seven days in culture (Figure 3e). In the following experiments, tooth development was analyzed in germs cultured on inserts.

**Table 2:** Tissues grown from tooth germs reconstructed with emtg-2 cells and EC (1:1~0) and MT on culture inserts

	Tooth	Non-tooth	Total
Positive Control ^*1^	13	−	13
Negative Control ^*2^	−	10	10
Emtg-2:EC = 1:1	44	−	44
Emtg-2:EC = 1:0.75	6	−	6
Emtg-2:EC = 1:0.5	26	−	26
Emtg-2:EC = 1:0.25	3	3	6
Emtg-2:EC = 1:0	−	16	16

*1: germs prepared with EC (E16.5) and MT; *2: germs prepared with only MT.

#### 2.1.3. Improvement of the Microenvironment Depends on the Mixing Ratio

**Figure 4:**
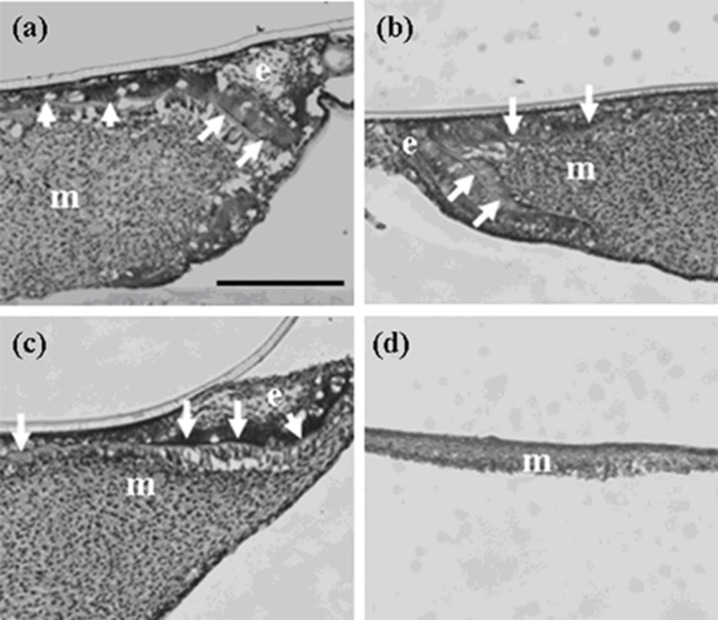
Histology of teeth developed from germs reconstructed with mixed epithelial cells (emtg-2 cells: EC = 1:0.75 to 0) and MT cultured for seven days on inserts. (**a**, **b**) All reconstructed germs (emtg-2 cells: EC = 1:0.75, 0.5) developed teeth. (**c**) Half of the germs (emtg-2 cells: EC = 1:0.25) developed teeth. Any histological difference was unrecognized among teeth developed from germs with different mixing rates (emtg-2 cells: EC = 1:0.75, 0.5 and 0.25). (**d**) No teeth developed from germs reconstructed with emtg-2 cells and EC (1:0) and MT. Only a thin layer of mesenchymal cells was recognized, e: epithelium, m: mesenchyme, arrows: very thin dentin-like structure, Scale bar is 100 μm in (**a**) and all figures are at the same magnification.

**Figure 5:**
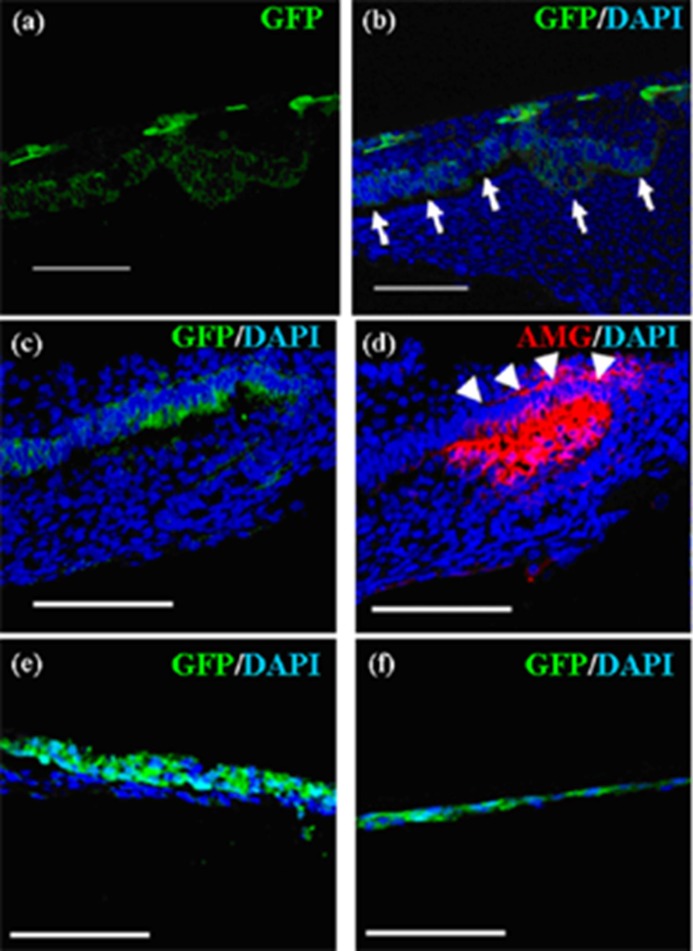
Localization of emtg-2 cells in constructed teeth. (**a**–**d**) Germs were prepared with GFP-labeled emtg-2 cells and EC (1:0.5) and MT, and cultured on inserts for seven days. Constructed teeth were subjected to immunohistochemistry. (**a**,**b**) GFP-labeled emtg-2 cells (green) were localized in the dental epithelium and inner enamel epithelium (arrows). (**c**,**d**) GFP-labeled emtg-2 cells (green) in the inner enamel epithelium expressed amelogenin protein (red) (arrowheads). (**c**) and (**d**) are serial samples. (**e**,**f**) Germs reconstructed with emtg-2 cells and MT were cultured for three and seven days, respectively. All scale bars are 100 μm. Nuclei were stained with DAPI (blue).

The addition of EC to the epithelial compartment of germs at 50% resulted in tooth construction at 100%. To analyze the mechanism in the improved construction, the mixing ratio was varied from 1:1 to 1:0. When the ratio was at 1:0.75 and 1:0.5, all germs developed teeth (Figure 4a,b and Table 2). When the ratio was lowered to 1:0.25, half of the germs developed teeth (Figure 4c and Table 2). The rate of tooth construction was decreased as the ratio was lowered. Elongated epithelial cells and a very thin dentin-like structure were detected in the constructed teeth (Figure 4 arrows). Germs reconstructed at 1:0 developed no teeth (Figure 4d and Table 2). In the germs, mesenchymal layers became very thin after three days and were undetectable after seven days in culture (Figure 5e,f).

#### 2.1.4. Developmental Stage of EC had Influence on Tooth Construction

EC were regularly prepared from dental germs at E16.5. To examine the effect of EC age, EC were prepared from embryos at E14.5, 15.5 and 16.5 and mixed with emtg-2 cells at 1:0.5. Germs reconstructed with the mixed epithelial cells and MT (E16.5) were cultured on inserts for seven days. Germs prepared with EC at different ages gave rise to varied rates of tooth development; 50% (E14.5), 86% (E15.5) and 100% (E16.5) (Table 3). 

**Table 3:** Tissues grown from tooth germs reconstructed with emtg-2 cells and EC (E14.5~16.5) and MT (E16.5) on culture inserts; emtg-2 cell: EC = 1:0.5

	Tooth	Non-tooth	Total
Positive Control ^*1^	4	−	4
Negative Control ^*2^	−	5	5
E14.5	3	3	6
E15.5	19	3	22
E16.5	6	−	6

*1: germs prepared with EC(E16.5) and MT; *2: germs prepared with only MT.

#### 2.1.5. Do Emtg Cells Actively Participate in Odontogenesis?

To examine whether emtg-2 cells contribute to the ameloblast layer, germs were prepared with GFP-labeled emtg-2 cells and EC (1:0.5) and with MT, and cultured on inserts. emtg-2 cells were detected intermittently in elongated epithelial cells adjacent to the mesenchyme (Figure 5a and b). Amelogenin protein was detected in cells in GFP-positive regions (Figure 5c and d) of serial section. The results demonstrated that emtg-2 cells were involved in the formation of the ameloblast layer and differentiated to ameloblasts.

**Figure 6:**
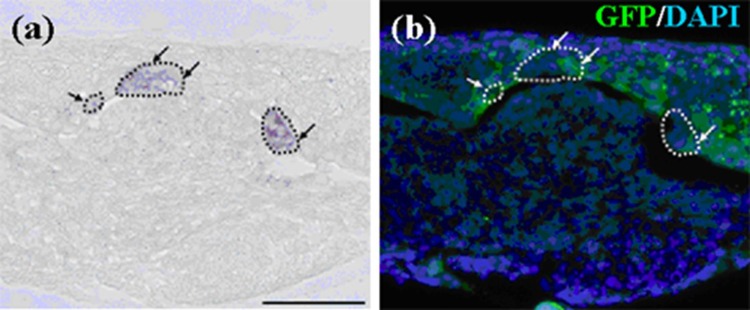
Shh expression in germs reconstructed with emtg-2 cells and EC (1:0.5) and MT and cultured for three days. (**a**) Shh expression (*in-situ* hybridization) was encircled with broken lines and pointed with arrows. (**b**) Immunohistochemistry with anti-GFP antibody localized emtg-2 cells (green) in the serial section, overlapping Shh expression. Scale bar is 100 μm and (**b**) is at the same magnification.

The enamel knot is believed to be a critical center of odontogenesis and is localized as a Shh-expressing spot [27]. *In-situ* hybridization demonstrated that Shh expression was detected in germs after three days in culture (Figure 6a). In addition, Shh-expressing cells were localized in GFP-labeled cell layers (Figure 6b) of serial sections, strongly indicating that some of emtg-2 cells were actively participating in odontogenesis as members of enamel knot-composing cells.

#### 2.1.6. Tooth Germs Were Reconstructed with Cells of Emtg-1 to -5 Lines and EC (1:0.5) and with MT

**Figure 7:**
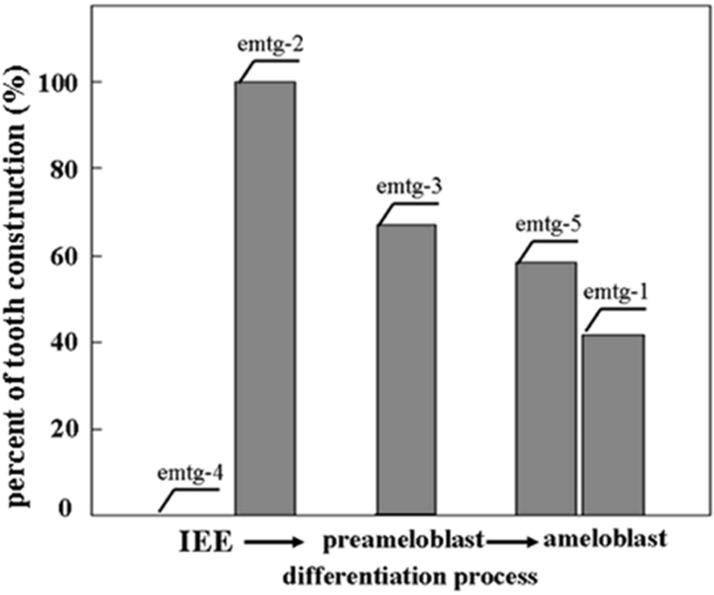
Schematic presentation of successful tooth construction rates of germs with emtg cell lines and EC (E16.5) at 1:0.5 and with MT (E16.5)in culture. Based on the result of RT-PCR analysis [18], emtg cell lines were lined up on the differentiation stage of ameloblast-lineage cells (http://bite-it.helsinki.fi/). The vertical line presents successful tooth construction (%) calculated from data presented in Table 4. IEE: inner enamel epithelium.

To examine whether the mixing effect with EC (E16.5) is observed on other emtg lines, germs were prepared with cells of each line (emtg-1 and -5; ameloblast-like cells, emtg-2 and -3; preameloblast-like cells, emtg-4; inner enamel epithelial-cell-like cells [18]) and EC at 1:0.5 and with MT (E16.5), and cultured for sevel days. As summarized in Figure 7, the rates of tooth construction varied among cell lines. Interestingly, none of germs with emtg-4 cells developed any tooth (Figure 7 and Table 4). 

**Table 4:** Tissues grown from tooth germs reconstructed with emtg (1 to 5) cells and EC (E16.5) and MT (E16.5) on culture inserts; emtg cell : EC = 1:0.5.

	Tooth	Non-tooth	Total
Positive Control *^1^	7	−	7
Negative Control *^2^	−	9	9
emtg-1	5	7	12
emtg-2	6	−	6
emtg-3	8	4	12
emtg-4	−	12	12
emtg-5	7	5	12

*^1^: germs prepared with EC and MT; *^2^: germs prpared with only MT.

#### 2.1.7. Gene Expression Patterns were Compared between Tooth Germs Prepared with and without EC.

On culture inserts, germs were reconstructed with a mixture of emtg-2 cells and EC (ratio = 1:1~0.5) and with MT developed teeth at 100%, but none of the germs without EC developed a tooth. In order to understand the microenvironments in the epithelial compartments of germs, gene expression patterns in emtg-2 cells adjacent to MT were compared between germs reconstructed with and without EC. Morphogenetic genes such as Shh, Lef1 are expressed for a relatively long period. Instead, the genes expressed transiently in germs would precisely reveal the stages of germs. Therefore, the following genes were selected: *Netrin3*, *Fgf9*, *Notch1*, *Oasis*, *N-myc*, *Dlx3*, *Bmp5* and *amelogenin* (Table 5) [28,29,30,31,32,33,34,35,36,37].

**Table 5:** Summary of gene expression pattern in the inner enamel epithelium at developmental stages (*in-situ* hybridization [38])

	initi.	bud	cap	bell	diff.	Sec.
*Netrin3*	+	−	−	−	N.D.	N.D.
*Fgf9*	+	+	−	+	−	−
*Notch1*	+	+	−	+	−	−
*Oasis*	−	−	+	+	+	−
*N-myc*	N.D.	N.D.	+	−	N.D.	N.D.
*Dlx3*	−	−	−	+	+	N.D.
*Bmp5*	−	−	−	−	+	+
*amelogenin*	N.D.	N.D.	N.D.	−	−	+

Note: initi.: initiation state; bud: bud state; cap: cap stage; bell: bell stage; diff.: differentiation stage; sec.: secretory stage; N.D.: not done.

**Figure 8:**
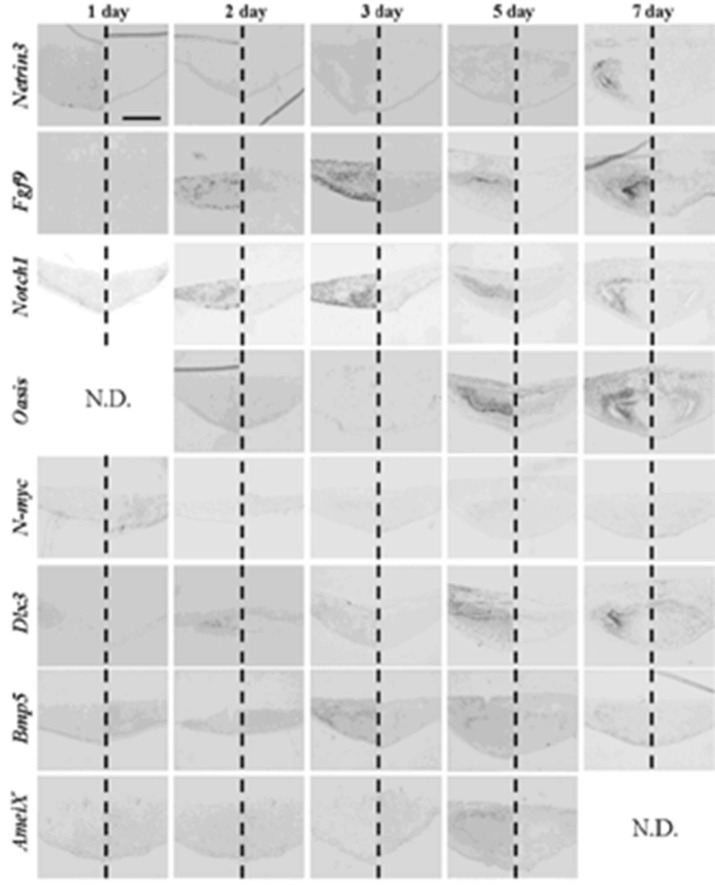
Gene expression pattern in germs reconstructed with EC and MT (positive control). Gene expressions were examined with *in-situ* hybridization with antisense probe in left and sense probe in right of each panel. Germs were cultured on inserts for one to seven days. Scale bar is 200 μm and all figures are at the same magnification.

**Figure 9:**
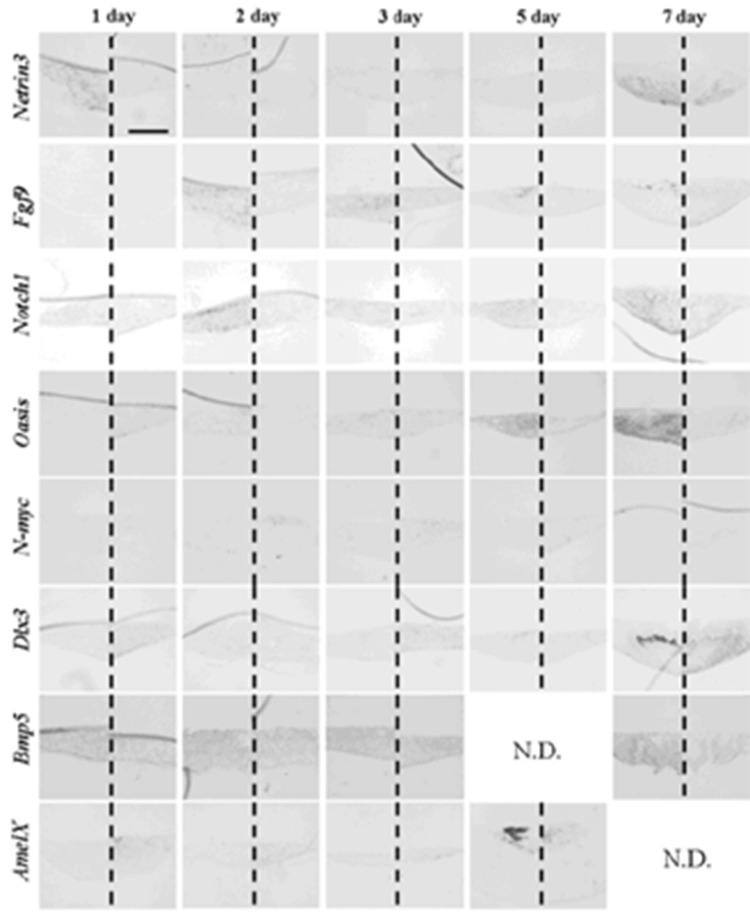
Gene expression pattern in germs reconstructed with emtg-2 cells and EC (1:0.5) and MT. Gene expressions were examined with *in-situ* hybridization with anti-sense probe in left and sense probe in right of each panel. Germs were cultured on inserts for one to seven days. Scale bar is 200 μm and all figures are at the same magnification.

After one to two days in culture, germs reconstructed with EC and MT (positive controls) took the same gene expression pattern as observed at the initiation stage (Figure 8 and Table 6). Germs reconstructed with mixed epithelial cells (emtg-2 cells: EC = 1:0.5) and with MT took a similar gene expression pattern as observed in positive controls (Figure 9, 12 and Table 6) with slight difference in *Bmp5* expression; after three days in culture, *Bmp5* expression was detected in positive controls, but it was undetectable in germs reconstructed with mixed epithelial cells. 

**Figure 10:**
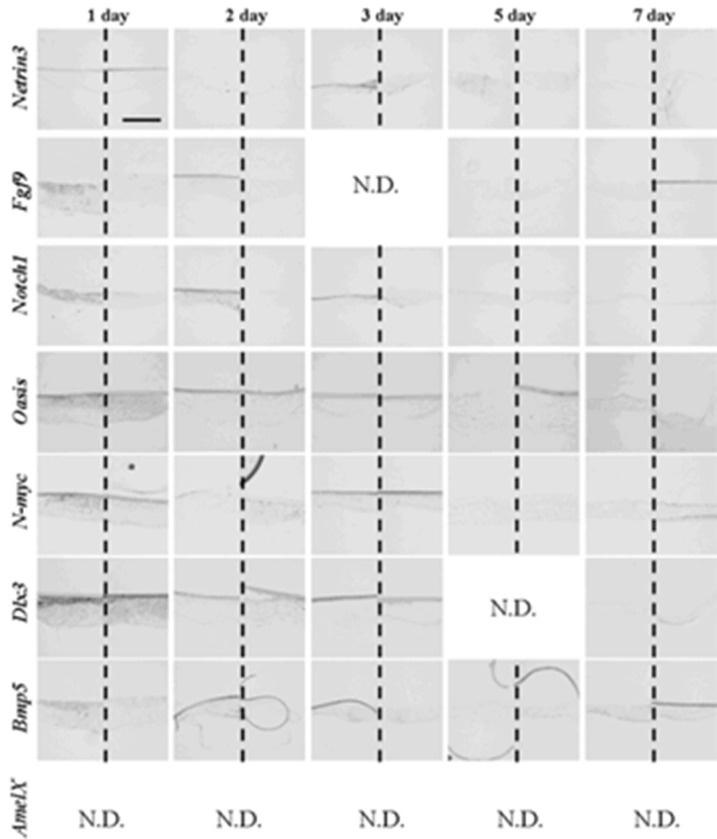
Gene expression pattern in germs reconstructed with emtg-2 cells and MT. Gene expressions were examined with *in-situ* hybridization with anti-sense probe in left and sense probe in right of each panel. Germs were cultured on inserts for one to seven days. Scale bar is 200 μm and all figures are at the same magnification.

**Figure 11:**
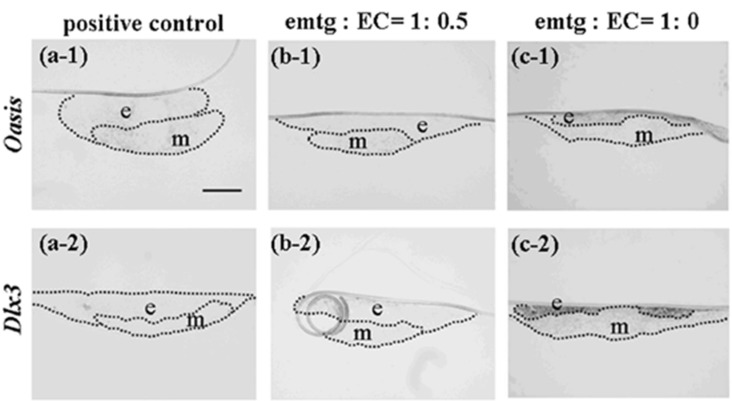
Expressions of *Oasis* and *Dlx3* at a high magnification in germs cultured for one day. (a-1, 2) *In-situ* hybridization in reconstructed germs with EC and MT (positive control). (b-1, 2) *In-situ* hybridization in reconstructed germs with emtg-2 cells and EC (1:0.5) and MT. (c-1, 2) *In-situ* hybridization in reconstructed germs with emtg-2 cells and MT. (a-1, b-1, c-1) Expression of *Oasis* in reconstructed germs. (a-2, b-2, c-2) Expression of *Dlx3* in reconstructed germs. e: epithelium, m: dental mesenchyme. Scale bar is 200 μm in (a-1) and all figures are at the same magnification.

After one day in culture, germs reconstructed with only emtg-2 cells and MT took the gene expression pattern similar to that of bell stage (Figure 10 and Table 6). Gene expressions were compared among three groups of germs (emtg-2 cells: EC = 0:1, 1:0.5, 1:0) (Figure 8, Figure 9, Figure 10, Figure 11, Table 6). Expressions of *Fgf9, Notch1, Oasis, N-myc* and *Dlx3* were decreased in epithelial cells of germs (emtg-2 cells: EC = 0:1, 1:0.5), but not in germs (emtg-2 cells: EC = 1:0).

**Table 6:** Summary of gene expression patterns in reconstructed germs with EC and MT (positive control), emtg-2 cells and EC (ratio = 1:0.5) and MT, and emtg-2 cells and MT. Germs were cultured for one to seven days.

Positive Control					
	1d	2d	3d	5d	7d
*Netrin3*	+	−	−	−	+
*Fgf9*	−	+	+	+	+
*Notch1*	−	+	+	+	+
*Oasis*	N.D.	−	−	+	+
*N-myc*	−	−	−	−	−
*Dlx3*	−	−	−	+	+
*Bmp5*	−	−	−	+	N.D.
*amelogenin*	N.D.	N.D.	N.D.	-	-
Emtg-2:EC = 1:0.5					
	1d	2d	3d	5d	7d
*Netrin3*	+	−	−	−	+
*Fgf9*	−	+	+	+	+
*Notch1*	−	+	+	+	+
*Oasis*	−	−	−	+	+
*N-myc*	−	−	−	−	−
*Dlx3*	−	−	−	+	+
*Bmp5*	−	−	−	N.D.	+
*amelogenin*	−	−	−.	+	N.D.
Emtg-2:EC = 1:0					
	1d	2d	3d	5d	7d
*Netrin3*	−	−	−	−	−
*Fgf9*	+	−	N.D.	−	−
*Notch1*	+	+	−	−	−
*Oasis*	+	−	−	−	−
*N-myc*	+	−	−	−	−
*Dlx3*	+	−	−	N.D.	−
*Bmp5*	−	−	−	−	−
*amelogenin*	N.D.	N.D.	N.D.	N.D.	N.D.

The *in-situ* hybridization analyses clearly demonstrated that addition of EC to the epithelial compartment changed the gene expression pattern of reconstructed germs, supporting the hypothesis that the dental program in emtg-2 cells was canceled by mixing with EC.

#### 2.1.8. Was the Reprogramming of emtg-2 cells Caused Directly by EC or Indirectly by EC Through MT?

**Figure 12:**
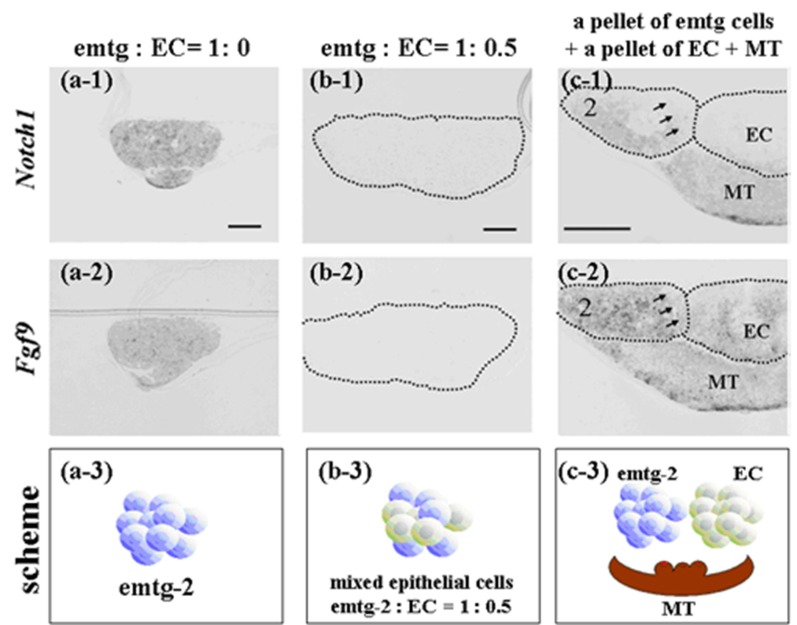
*In-situ* hybridization of *Notch1* and *Fgf9* in germs reconstructed with a modified method and cultured for one day. (**a-1**, 2) Gene expression in a pellet of emtg-2 cells. (**b-1**, 2) Gene expression in a pellet of emtg-2 cells and EC (1:0.5). (**c-1**, 2) Gene expression in reconstructed germs with a pellet of emtg-2 cells and a pellet of EC and MT. (**a-1**, **b-1**, **c-1**) Expression of *Notch1* in reconstructed germs. (**a-2**, **b-2**, **c-2**) Expression of *Fgf9* in reconstructed germs. (**a-3**, **b-3**, **c-3**) Illustration of the modified method. 2: emtg-2 cells, EC: dental epithelial cells, MT: dental mesenchyme. Scale bars are 100 μm in (**a-1** and **b-1**) and (**a-1**, **a-2**, **b-1** and **b-2**) are at the same magnification. Scale bar is 200 μm in (**c-1**) and (**c-2**) is at the same magnification.

It is well established that tissue interactions between epithelial and mesenchymal compartments drive organogenesis. In addition, cellular interactions in each compartment may be critical in odontogenesis. To examine whether EC have direct effects on gene expression of emtg-2 cells, dental germs were prepared with a modified method; the epithelial compartment was composed of a pellet of emtg-2 cells (1.5 × 10^4^ cells) and a pellet of EC (1.5 × 10^4^ cells), sitting in parallel on MT. In a regular germ, a pellet containing homogeneously the two epithelial populations (3 × 10^4^ cells/germ) was sitting on MT (See Figure 12 a-3, b-3, c-3). The modified germs were cultured for one day and subjected to *in situ* hybridization analysis. 

**Figure 13:**
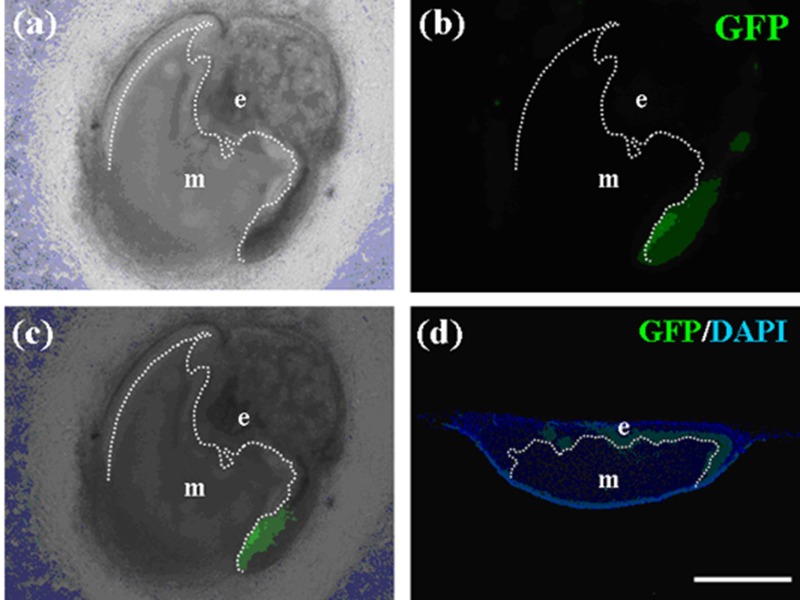
Immunohistochemistry of teeth developed from germs cultured for seven days. (**a**) Bright field photo of a constructed tooth with a pellet of GFP-labeled emtg-2 cells and a pellet of EC and with MT. (**b**) Immunohistochemistry with anti-GFP antibody detected GFP-labeled cells in an enamel organ (green). (**c**) (**a**) and (**b**) were marged. GFP-labeled emtg-2 cells were detected in cervical loop area. (**d**) Immunohistochemistry with anti-GFP antibody detected GFP-labeled cells in epithelium at right side in the section of (**a**). Nuclei were stained with DAPI (blue) in (**d**). dot line; basement membrane, e: epithelium, m: mesenchyme. Scale bar is 500 μm in (**d**) and all figures are at the same magnification.

Expressions of *Notch1* and *Fgf9* were detected in pellets containing only emtg-2 cells (Figure 12a), but not in pellets containing emtg-2 cells and EC (1:0.5) (Figure 12b). Interestingly, in the modified germs, expressions of *Notch1* and *Fgf9* were reduced in emtg-2 cells facing the EC (Figure 12c), but not in emtg-2 cells facing MT, and emtg-2 cells were localized in a right cervical loop area after seven days in culture (Figure 13). These observations suggested that cancellation of the developmental program of emtg-2 cells was induced directly by EC.

### 2.2. Discussion

The significance of “niche” or “microenvironment” has been often emphasized, especially in developmental biology, but it was used as an abstract argument and an obstacle against analytical biology. However, recent technical advancement and the movement of “tissue engineering” have made the concept of niche a much more practical theme. Several recent studies indicate that a microenvironment cancels the already-made program in foreign cells and allows them to take its own program [19,20,21]. 

Our previous study reported that five clonal emtg lines expressed sets of genes specific for different developmental stages of the dental epithelium and cells of emtg-2 expressed genes specific for preameloblasts [18]. The present study demonstrated that reconstructed germs developed into teeth at 100% when emtg-2 cells in the epithelial compartment were mixed with EC (E16.5) (> 1:0.5). The epithelial compartment in the improved germs revealed the pattern of gene expression which is observed at the initiation stage corresponding to E11.5–12.5 [1,2]. Enzymatic separation and recombination of the epithelial and mesenchymal compartments set the developmental clock back to the initiation stage [22,23,24,25,26]. These observations suggest that the developmental program in emtg-2 cells was canceled by the microenvironment created in the germs. Then, the microenvironment might coordinate two populations of epithelial cells and mesenchymal cells and allow them to restart odontogenesis from the initiation stage. 

The *in vitro* model on culture insert is useful, although it has several weaknesses that need to be improved upon. One of them is the extra-formation of enamel knots or Shh-expression spots. After three days in culture, reconstructed germs with cells of emtg-2 and EC expressed the pattern of gene expression similar to that of bud stage, and three Shh-expression spots were detected in the germs. Another weakness is poor formation of basement membrane. The amelogenin expression was appropriate in the germ implanted under kidney capsule [18]. However, the boundary between epithelial and mesenchymal compartments became less prominent when the germs were cultured on inserts instead of implanting under kidney capsule. It is possible that the formation of the basement membrane is not fully progressing and secreted amelogenin protein spreads into the area of mesenchyme.

In our previous study, germs reconstructed with only emtg-2 cells and MT developed into tooth at near 60% when they were implanted under kidney capsule [18]. In the present study, however, germs reconstructed with only emtg-2 cells and MT developed into no teeth when they were cultured on inserts. In general, the environment under kidney capsule is known to be superior to those of any tissue culture dishes. It is reasonable to think that the undefined factors under kidney capsule help the reconstructed germs to cancel the program in emtg-2 cells and allow them to restart odontogenesis from the initiation stage. 

Germs reconstructed with mixed epithelial cells (emtg cells: EC = 1:0.5) and MT developed into teeth at varied efficiency: 42% (emtg-1), 100% (emtg-2), 67% (emtg-3), 0% (emtg-4) and 58% (emtg-5). A similar but not identical tooth construction was observed when germs were reconstructed with cells of each emtg line and MT and implanted under kidney capsule [18]. In both studies, MT were regularly prepared from embryos at E16.5. In addition, germs prepared with EC at different ages developed into to teeth at varied rates; 50% (E14.5), 86% (E15.5) and 100% (E16.5) (Table 3). These observations indicate that the age of tissue (cells) and cell lines in reconstructed germs has critical influence on tooth construction. Heterochronological recombination of dissociated epithelial and mesenchymal cells disturb tooth organogenesis [22], although the organogenesis progressed when tissue architectures of both epithelial and mesenchymal compartments were preserved [39].

In order to investigate how reprograming of emtg-2 cells is induced, germs were prepared with two pellets of epithelial cells. The results indicate that EC had direct effects on emtg-2 cells. If this is the case, more EC in germs would give rise to higher tooth construction. As expected, the rate of successful tooth reconstruction was decreased as the mixing ratio was reduced from 1:1 to 1:0 (emtg-2: dental epithelial cells). Germs reconstructed with emtg cells and MT might have some difficulty to create the microenvironment which coordinates epithelial and mesenchymal components to restart odontogenesis from the first stage of a series of reciprocal epitheo-mesenchymal interactions [1,2]. “EC give emtg cells direct signals, diffusible factors and/or physical contact, which are highly expressed at E16.5, then the epithelial compartment becomes competent to communicate with the mesenchymal compartment. To identify the signals would further help to understand odontogenesis.”

## 3. Materials and Methods

### 3.1. Animals

CD-1 mice (Charles River Japan, Yokohama, Japan) were maintained in the experimental animal facility of Tokyo University of Science. They were kept under a 12:12 h light: dark cycle at 22–24 °C. Standard laboratory feed (MR standard, Nousan LTD, Yokohama, Japan) and tap water were given *ad libitum*. Mice care and handling conformed to the NIH guidelines for animal research. The Institution Animal Care and Use Committee approved the experimental protocols.

### 3.2. Cell Culture

The medium was a 1:1 mixture of Dullbecco’s modified Eagle’s medium and Ham’s nutrient mixture F12 (DMEM/F12, Sigma, St. Louis, MO, USA) supplemented with heat-inactivated fetal calf serum at 10% (10% FCS v/v, Sigma, St. Louis, MO, USA), penicillin (31 ìg/mL, Sigma, St. Louis, MO, USA), streptomycin (50 ìg/mL, Sigma, St. Louis, MO, USA), insulin (10 ìg/mL, Sigma, St. Louis, MO, USA) and transferrin (10 ìg/mL, Sigma, St. Louis, MO, USA). Cell lines were maintained in the medium and passaged at 10% on 100 mm dishes (FALCON; Becton Dickinson Labware, Franklin Lakes, NJ, USA).

### 3.3. Histology and Immunohistochemistry

For histology, samples were fixed in 4% formaldehyde at 4 °C overnight and dehydrated with sucrose for frozen sections, and embedded in O.C.T. Compound (Sakura Finetek, Torrance, CA, USA), or dehydrated with ethanol for paraffin embedding. Samples were cut into 10 ìm sections. Sections were stained with hematoxyline and eosin, or incubated with antibodies. The primary antibodies used were anti-GFP (1:500, Santa Cruz Biotechnology, Inc., Heidelberg, Germany) and anti-amelogenin (1: 100, Hokudo Co., Ltd., Sapporo, Japan) rabbit polyclonal antibodies. After several washes with PBS containing 0.1% Tween-20 (Sigma, St. Louis, MO, USA), sections were incubated with secondary antibodies; Alexa Fluor 488 goat anti-rabbit IgG (1:500, Invitrogen Corp., CA, USA) and Alexa Fluor 594 goat anti-rabbit IgG (1:500, Invitrogen Corp., CA, USA) for 1.5 h at room temperature. Negative controls were incubated without primary antibodies. Nuclei were stained with DAPI.

### 3.4. Preparation of Bioengineered Tooth Germs

**Figure 14:**
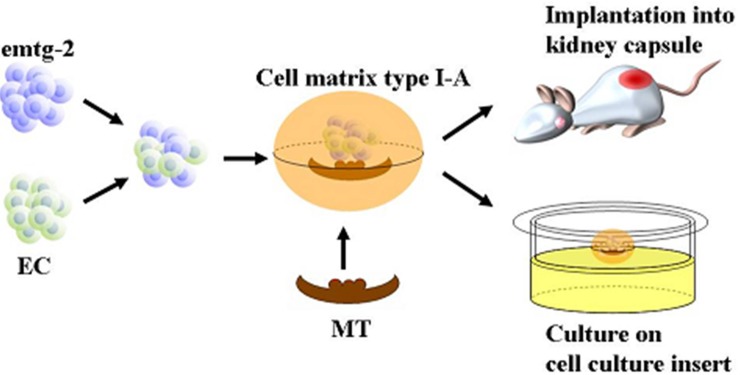
Preparation of artificial tooth germ.

The method was developed and described elsewhere [22]. In brief, dental germs of lower first molars were dissected from embryos at day (E) 14.5, 15.5 and 16.5 and incubated in PBS containing 1000 U/mL dispase II (SANKOJUNYAKU CO. LTD., Tokyo, Japan) for 15 min at room temperature, and the epithelia were separated from the mesenchymal tissues with a fine needle. Epithelial fractions were treated with 100 U/mL collagenase type I (Worthington Biochemical Co., Lakewood, NJ, ISA) for 20 min at 37 °C. After several washings with PBS, they were treated with 0.5 × trypsin-0.53 mM EDTA (Sigma, St. Louis, MO, USA) for 5 min at 37 °C and dispersed. emtg cells and dental epithelial cells were mixed at ratios of 1:1, 1:0.75, 1:0.5, 1:0.25 or 1:0. Suspensions containing epithelial cells were centrifuged to make pellets. The total epithelial cell number was 3 × 10^4^ cells/germ. Tooth germs were reconstructed with the pellets of mixed epithelial cells and with dental mesenchymal tissues prepared from embryos in 30 ìL Cell matrix type I-A (Nitta Gelatin Inc., Osaka, Japan) and incubated in a humidified atmosphere of 95% air and 5% CO2 at 37 °C. On the following day, the reconstructed tooth germs were implanted under kidney capsule or cultured on cell culture inserts (FALCON; Becton Dickinson Labware, Franklin Lakes, NJ, USA) for seven days (Figure 14).

### 3.5. GFP Infection with Retrovirus Vector

Plat-E packaging cells were transiently transfected with pMXs-Ig modified at 5'LTR to LTR/CMV (gifted from Dr. Murakami, Tokyo University of Science), using LipofectamineTM2000 Reagent (Invitrogen). After 48 h, the transfection solution was added to each emtg cell line cultured on 24-well plates.

### 3.6. *In-Situ Hybridization* should it be In-Situ in the second title without italic, I am not sure the Cell rule

Samples were embedded in O.C.T. Compound (Sakura Finetek) without fixation and frozen. Cryosections (10 ìm) were made and mounted on FRONTER slides (MATSUNAMI, Osaka, Japan). Digoxigenin-labeled probes for specific transcripts were produced with the digoxigenin RNA labeling kit (Roche, Mannheim, Germany) and prepared by PCR with primers designed using published sequences (Table 7 and [22]). Resultant bands were subcloned into pGEM T-easy vector (Promega, Madison, WI) and sequenced to confirm the sequence identity. Hybridization was carried out at 57 °C for 16 h with hybridization buffer containing 50% formamide, 0.01 M Tris-HCl (pH 7.4), 1 mM EDTA, 0.1% SDS, 0.6 M NaCl, 1× Denhardt’s solution, 10% dextran sulfate, and 400 ìg/mL yeast tRNA. The final wash was in 0.2× SSC at 57 °C for 1 h. Samples were incubated for 2 h with alkaline phosphatase-conjugated sheep anti-digoxigenin Fab fragments (Roche) at 1:1000, washed and processed for colorimetric detection using NBT/BCIP Stock Solution (Roche).]

**Table 7:** Primers of marker gene’s probe

Gene	Left primer	Right primer	Length of probe
*Netrin3*	ccttctctgggctctgtgtc	tgggagtagcttgctgacct	612 bp
*Fgf9*	agctccactgttgccaaact	tgggctatgataccagtgtga	419 bp
*Notch1*	agagatgtgggatgcaggac	tttggatgatgctgtttgga	844 bp
*Oasis*	tatccgtcctcctccgataa	aaagcatgtgctgagctggt	409 bp
*N-myc*	tttcaaattggtcccctgtc	acccagttctatgcaccaaa	702 bp
*Dlx3*	tgtccttttccaaggacctg	tccttcacttcccacgaaac	710 bp
*Bmp5*	tctgtgctatgtttatgaccacaat	ggtttttaaaattttggttgttagtt	404 bp
*AmelX*	taccacctcatcctggaagc	ggagaacagtggaggcagag	449 bp

## 4. Conclusions

Bioengineered germs prepared with emtg cell lines develop teeth at 100% rate when emtg cells are mixed with primary embryonic epithelial cells in the epithelial compartment. The primary epithelial cells in the compartment help emtg cells to communicate and to initiate odontogenesis with the mesenchyme. 
